# Thoracoscopic Diagnosis of Follicular Lymphoma Relapsing after 13 Years

**DOI:** 10.1055/s-0038-1661416

**Published:** 2019-02-14

**Authors:** Serda Kanbur, Onur Derdiyok, Hakan Kiral, Hakan Yilmaz, Mine Demir, Cagatay Tezel, Volkan Baysungur, Serdar Evman

**Affiliations:** 1 Department of Thoracic Surgery, Sureyyapasa Chest Diseases and Thoracic Surgery Training and Research Hospital, Istanbul, Turkey

**Keywords:** follicular lymphoma, thoracic surgery, relapsing after 13 years

## Abstract

Relapse in lymphoproliferative malignancies is not an exceptional entity and generally occurs within the first 2 or 3 years following the primary treatment. Lymph node biopsy is essential for the diagnosis of relapse and treatment. A 64-years-old woman was referred to our clinic for back pain and dyspnea. Chest X-ray and computed tomography (CT) showed pleural thickening in the right hemithorax and pleural effusion. Hereby, we report a patient with a history of follicular lymphoma treatment 13 years ago, presenting with unilateral pleural effusion and being diagnosed, unpredictably, with relapsing lymphoma by video-assisted thoracoscopic surgery pleural biopsy.

Chest wall derived lymphoma is often accompanied by pleural effusion or chylothorax. Primitive malignant lymphoma originating from chest wall constitutes 0.3% of extranodal lymphoma. Symptoms often include fever, fatigue and weight loss, and extranodal involvement such as skin, lungs, pleural effusion, stomach, and tonsils. Malignant lymphoma is very rare when there is no previous inflammatory disease in recurrent pleural effusions. In this study, our patient who had received chemotherapy for lymphoma 13 years ago presents with video-assisted thoracoscopic surgery (VATS) due to recurrent pleural effusion in the context of our case report as a recurrent follicular lymphoma biopsy.

## Case Report

**Fig. 1:**
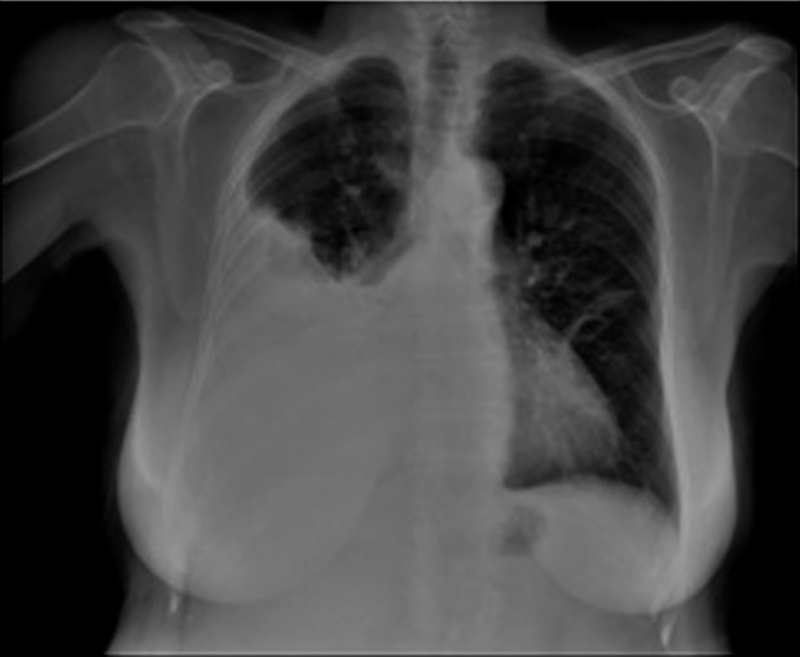
Posteroanterior chest X-ray showed increase in density in the lower zone of the right hemithorax .

A 64-years-old female patient was admitted to our clinic with complaints of shortness of breath. On physical examination, body temperature was 36.8°C, pulse was 150 per minute, respiratory rate was 24 per minute, and arterial blood pressure was 140/85 mm Hg. A decrease in respiratory sounds in the right lower zone with auscultation was present. Posteroanterior chest X-ray revealed pleural effusion in the lower right hemithorax ( Fig. 1 ). Her medical history included congestive heart failure treatment for 5 years, hysterectomy 25 years ago, and chemotherapy due to follicular lymphoma 13 years ago.

**Fig. 2:**
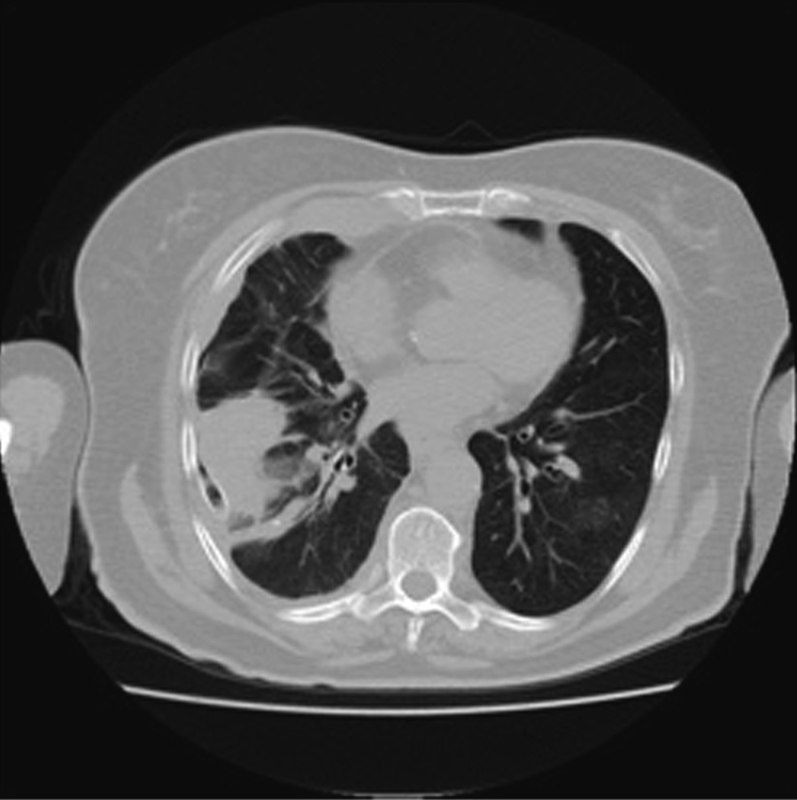
Computed tomography (CT) showing pleural thickening in the right hemithorax and pleural effusion.

**Fig. 3:**
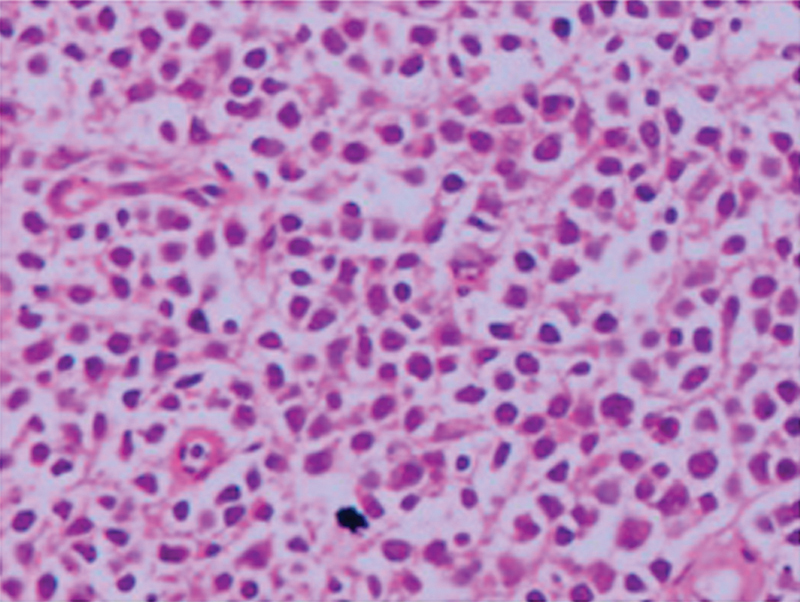
Biopsy specimen of the lower right parietal pleura. Megakaryocytes (hematoxylin and eosin staining; original magnification: × 40).

Computed tomography (CT) and positron emission tomography/CT (PET-CT) revealed pleural effusion and thickening, with a high maximum standard uptake value of 10.4 in the right hemithorax ( Fig. 2 ). Thoracentesis was performed with ultrasonography guidance. Biochemical examination of the liquid revealed albumin = 2.1 g/dL (blood: 2.9), total protein = 3 g/dL (blood: 7.1), and LDH = 460 U/L (blood:226), with 33% lymphocytes versus 66% leukocytes. The ARB of the liquid was negative. No endobronchial lesion was detected in fiberoptic bronchoscopy. Sputum and bronchoscopic lavage cultures were also negative. Subsequent to the cytological examination revealing atypical cells with no definite diagnosis, the patient underwent a VATS pleural biopsy. She was discharged on postoperative day 2, uneventfully. Definite pathology was reported as follicular lymphoma ( Fig. 3 ), and the patient was referred to the Hematology Department for further treatment. The patient only had chemotherapy treatment. No pathology was observed in 17-month follow-ups.

## Discussion

Lymphoproliferative malignancies frequently involve extranodal involvement with lymph node involvement and spleen involvement. Pleural invasion is reported in 30% of autopsies of lymphoma patients, and only 13% of all malign pleural effusions are lymphomas. 1 All tumors that are close to the pleural space or have metastasizing properties may cause pleural effusion. Approximately 40 to 50% of malignant pleural effusions are because of lung cancers. However, malignant pleural effusions may be seen in the course of breast cancer, ovarian cancer, Hodgkin's lymphoma, non-Hodgkin's lymphoma, gastric cancer, hepatic tumors, pancreatic cancer, and carcinoid tumors. 2 The incidence of pleural effusion complicated by noncoexisting lymphoma is between 6 and 19%. T-cell lymphomas are more often associated with pleural effusion than B-cell lymphomas. Pleural involvement in the majority of cases is detected as a finding of systemic disease. Pathophysiology of lymphoma effusions may occur secondary to impaired lymphatic drainage, pleural or pulmonary infiltration, venous occlusion, pulmonary infection, or radiation therapy. 3 The observation of 34 cases of lymphoma diagnosed by pleural biopsy revealed that 85.3% of the cases could be classified according to the World Health Organization lymphoma criteria. As for the invasion of lymphoma in pleural effusion M.D. Anderson Cancer Center Hospital, he suggested that the most common subtype in the study was aggressive lymphoma, including diffuse large B-cell lymphoma. 4

The presence of pleural effusion in patients with follicular lymphoma was found to be one of the risk factors that adversely affected survival. Among B-cell lymphomas, the follicular lymphoma is the second most common type of lymphoma with a frequency of 20% after diffuse large B-cell lymphoma (60%). Pleural effusion due to lymphoma suggests that the tumor burden is high. Five-year survival rates for high-risk follicular lymphoma patients range from 20 to 53%, depending on the prognostic index score used. 5 Treatment of the primary disease usually improves the pleural effusion. In high-risk symptomatic follicular lymphoma cases, rituximab-based chemotherapy has emerged at higher complication rates. In addition, in the treatment of rituximab, progression-free survival increased from 58 to 75% over 36 months. 5

VATS is a must in cases where the cause of pleural effusion cannot be determined. CT-guided pleural biopsy, as well as open pleural biopsy or thoracoscopy, yields clear results. But VATS, however, allows evacuation of the effusion with concomitant chemical pleurodesis and complete palliative treatment, as well as obtaining larger tissue for further studies that may be needed, with placement of a single pleural catheter at the same time. 6

Paramalignant pleural effusions caused by the local and systemic effects of the tumor, and the effects of radiotherapy and chemotherapy administrated should not be overlooked. When thoracentesis fails to provide a definitive diagnosis, VATS is indicated for both diagnostic and therapeutic purposes.

In conclusion, It should be kept in mind that lymphoma may be a very rare etiological factor in pleural effusions. Follicular lymphomas rarely occur as recurrent, lymphocytic pleural effusion without any other organ involvement.
